# COVID-19 breakthrough infections and humoral immune response among BNT162b2 vaccinated healthcare workers in Malaysia

**DOI:** 10.1080/22221751.2022.2065936

**Published:** 2022-05-03

**Authors:** Su Lan Yang, Adiratna Mat Ripen, Chin Tho Leong, Jen Ven Lee, Chia How Yen, Avinash Kumar Chand, Karina Koh, Nur Aisyah Binti Abdul Rahim, Varaalakshmy Gokilavanan, Nik Nur Eliza binti Mohamed, Raj Kumar A/L. Sevalingam, Nadirah Sulaiman, Ahmad Kamil bin Ab Razak, Nurul Haslinda binti Mohd Nor, Mei Kuan Pong, Ket Yan Tai, Valerie Toh, Yuan Liang Woon, Kalaiarasu M. Peariasamy

**Affiliations:** a Centre for Clinical Epidemiology, Institute for Clinical Research, National Institutes of Health, Ministry of Health Malaysia, Shah Alam, Malaysia; b Allergy & Immunology Research Centre, Institute for Medical Research, National Institutes of Health, Ministry of Health Malaysia, Shah Alam, Malaysia; c Clinical Research Centre, Hospital Kuala Lumpur, Ministry of Health Malaysia, Kuala Lumpur, Malaysia; d Clinical Research Centre, Hospital Queen Elizabeth II, Ministry of Health Malaysia, Kota Kinabalu, Malaysia; e Occupational Health Department, Hospital Queen Elizabeth, Ministry of Health Malaysia, Kota Kinabalu, Malaysia; f Clinical Research Centre, Hospital Queen Elizabeth, Ministry of Health Malaysia, Kota Kinabalu, Malaysia; g Institute for Clinical Research, National Institutes of Health, Ministry of Health Malaysia, Shah Alam, Malaysia

**Keywords:** COVID-19, breakthrough, antibody, BNT162b2, healthcare worker, vaccine, humoral immunity, IgG assay

## Abstract

The evaluation of breakthrough infection and humoral immunity responses are important outcomes for vaccination policy for healthcare staff. This prospective cohort study collected blood samples at 5-time points; before primary vaccine doses, and at 2, 10 and 24 weeks after BNT162b2 vaccination from 551 HCWs, between March and October 2021. We investigated the association between anti-spike-1 protein receptor-binding domain (anti-S1-RBD) antibody geometric mean titre (GMT) and breakthrough infections. Two weeks post-vaccination, the GMT of anti-S1-RBD antibodies was measured at almost maximum detectable value (3115 BAU/ml [95% CI, 3051–3180]); it decreased to 1486 BAU/ml (95% CI, 1371–1610) at 10 weeks; and to 315 BAU/ml (95% CI, 283–349) at 24 weeks. Prior COVID-19 infection and age significantly affected the antibody titres. Fifty-six participants, none of whom were COVID-19 convalescents, had breakthrough infections between 10 and 24 weeks post-vaccination. Before breakthrough infections, the GMT was not different between the breakthrough and non-breakthrough individuals. After infection, the GMT was significantly higher in individuals with breakthrough infections (2038 BAU/ml [95%CI, 1547–2685]), specifically in symptomatic breakthroughs, compared to those without infection (254 BAU/ml [95%CI, 233–278]). A notable surge in breakthrough infections among healthcare workers coincided with the emergence of the Delta variant and when BNT162b2-elicited antibody responses waned in 10–24 weeks (i.e. approximately 3–6 months). Post-breakthrough, the antibody response was boosted in individuals with symptomatic presentations, but not asymptomatic individuals. The study finding supports administering booster vaccination for healthcare staff, including those who recovered from asymptomatic breakthrough infection.

## Introduction

Two years into the pandemic, COVID-19, caused by severe acute respiratory syndrome coronavirus 2 (SARS-CoV-2), is responsible for over 493 million infections and over 6 million deaths [1]. Vaccination and public health and social measures have become key strategies for controlling the pandemic [2,3]. Vaccines play a critical role in preventing severe outcomes [3]. In Malaysia, BNT162b2 (Pfizer-BioNTech) mRNA vaccine was the first to be included in the national vaccination programme in February 2021. As of January 2022, 88.3% of the Malaysian population (age 12 years and above) has completed their primary vaccination series. The vaccines used include Pfizer/BioNTech (57.8%), Sinovac (33.7%), AstraZeneca (8.1%), and CanSinoBio (0.3%) vaccine [4].

Clinical trials and real-world data demonstrated the high effectiveness of BNT162b2 against severe COVID-19 and deaths [5–9]. These findings notwithstanding, countries worldwide are facing the new threats of the highly infectious SARS-CoV-2 Omicron variant, thus a need for on-going re-evaluation of the immunity protection in the context of emerging variants. Growing evidence has shown vaccine effectiveness declines with time since vaccination and reduced neutralizing capacity against a newer variant of concern (VOC) [10]. However, the degree of waning varies by vaccine products, population vaccination coverage, the extent of natural infection, at-risk population, and circulating virus strains [11]. These factors are important for the consideration of implementing booster vaccines. To strive for global equity in vaccine access, WHO advised against the blanket roll-out of boosters to all populations but instead to use a tailored approach for booster policy based on the epidemiology of breakthrough infections, time since vaccination, and at-risk population, and to supplement with immunogenicity studies of the vaccines in use [11]. Therefore, country-specific longitudinal monitoring of clinical and immunology protection is crucial to inform the necessity and timing of booster doses as part of a country’s vaccination policy.

Healthcare workers (HCWs) are at a 3.4 times higher risk of testing positive for COVID-19 than the general community due to their occupational exposure from direct patient contact and the availability of personal protective equipment (PPE) [12]. Preserving the health of HCWs is essential to protect the health system, and hence HCWs were prioritized for BNT162b2 when it was first made available in Malaysia. We studied the humoral responses and breakthrough infections in this high-risk group to understand the immunology and clinical protection of BNT162b2 in a racially-diverse Malaysian population.

In this study, we undertook surveillance of HCWs for occupational risk, information on any COVID-19 symptoms, including reverse-transcriptase polymerase chain reaction (RT–PCR) test results, and serum analysis for humoral response with BNT162b2 vaccination. Specifically, the objective was to estimate the geometric mean titre (GMT) of antibodies against the anti-spike-1 protein receptor-binding domain (anti-S1-RBD) amongst BNT162b2 recipients up to 24 weeks after vaccination for the history of any breakthrough infection.

## Materials and methods

### Study design and population

This prospective, single-arm cohort study was conducted in Malaysia between March and October 2021. Five hundred and fifty-one healthcare workers, who received two doses of BNT162b2 vaccines three weeks apart, were recruited from three tertiary public hospitals, of which two were designated for the management of COVID-19 patients. Participant recruitment was by quota sampling considering the population of the healthcare staff in each hospital. The study was approved by the Medical Research and Ethics Committee (MREC) Ministry of Health Malaysia and registered (NMRR-21-56-58212). All participants provided written informed consent before enrolment.

### Sample collection and self-administered questionnaire

Blood samples were collected at 5 scheduled time points – before the first dose of vaccination (pre-vac 1), before the second dose (pre-vac 2), and at 2, 10 and 24 weeks after the second dose. Participants who missed any scheduled appointment were allowed to attend subsequent visits.

Participants’ socio-demographic data, comorbidities and history of COVID-19 infection were obtained at baseline. All HCWs completed a self-administered questionnaire to assess exposure to SARS-CoV-2 and adherence to infection control measures at the workplace, and compliance to public health and social measures in the community (Supplementary material). All study data were recorded and managed using the research electronic data capture (REDCap) platform [13].

### Breakthrough infection reporting

An automated instant message was delivered to participants every two weeks to prompt updates on the presence of COVID-19-related symptoms. For any participant whose symptoms fulfilled the clinical criteria of suspected COVID-19 infection, an RT–PCR test was performed, and the result was documented. Asymptomatic breakthrough infections detected through contact tracing and institutional surveillance were also recorded.

### Serology assay

Blood samples were centrifuged for serum separation on the day of collection at the site and kept in multiple cryovials in a −80°C freezer until transport to the Institute for Medical Research (IMR) central laboratory. Temperatures were monitored closely at 2–8°C during transportation. All samples were tested for IgG antibody against SARS-CoV-2 spike protein (S1) receptor-binding domain (RBD) with ADVIA Centaur SARS-CoV-2 spike IgG assay, sCOVG (Siemens Healthcare Diagnostic, NY, USA). The sCOVG assay is a fully automated two-step sandwich immunoassay using indirect chemiluminescent technology for qualitative and quantitative detection of IgG, including neutralizing antibodies [14]. The assay has a specificity of 99.4–99.9% and sensitivity of 90.5–91.14% and is highly correlated (Spearman’s r = 0.843) to plaque reduction neutralization titre (PRNT_50_) [14,15]. The assay outputs an sCOVG index value (U/ml) and can be standardized to WHO International Standard for anti-SARS-CoV-2 Immunoglobulin following the conversion rate “sCOVG index value*21.8 = BAU/ml” [14]. The seropositivity cut-off is 21.8 BAU/ml (1 U/ml), and the upper quantification threshold is 3270 BAU/ml (150 U/ml). All samples were processed according to the manufacturer’s procedures by trained laboratory personnel.

### Statistical analysis

The anti-S1-RBD IgG antibody titre was summarized in a geometric mean and 95% confidence interval. Antibody titre at each timepoint, stratified by covariates, was compared using Mann–Whitney U test for covariates with two groups, or Kruskal–Wallis nonparametric test and followed by Dunn’s post -hoc test for covariates with more than two groups. Statistical significance was defined when *p *< 0.05.

We used a linear mixed model to evaluate the covariates associated with the change of antibody titre over 24 weeks. The dependent variable was the anti-S1-RBD antibody titre. The random effect was individual participant level. The fixed-effect covariates tested were time, 10-year age groups, sex, body mass index (BMI), history of COVID-19 infection, comorbidity and occurrence of breakthrough infection during the follow-up period. Time was modelled as a categorical variable (pre-vac 1, pre-vac 2, 2-weeks, 10-weeks, and 24-weeks). Covariates and interaction terms were kept in the model if the *p*-value was <0.05, and it improved the model fit based on Akaike Information Criterion (AIC). To use the linear mixed model, missing data on dependent variables (antibody titre) at various time points were assumed to be “missing at random,” while two participants with missing data on covariates were excluded from the analysis. A residual plot and normal Q-Q plot were used to assess the model assumption. The details of the linear mixed model are presented in Supplementary Table 1. All analysis and data visualization were performed using R software, version 4.1.1 (the R Foundation for Statistical Computing). The linear mixed model was analyzed using the lmer and lmerTest package in R.

## Results

### Baseline demographic characteristics

The demographic characteristics of the 551 participants are provided in Table 1. All major ethnic groups in Malaysia are represented in the study population. The mean (SD) age was 33.7 (7.2) years, 417 (75.7%) of them were female and the mean BMI was 27.1(5.8) kg/m^2^. Most (*n* = 452, 82%) of our participants were healthy without comorbidities; the most common self-reported comorbidities were hypertension (*n* = 34, 6.2%), allergy (*n* = 28, 5.1%), respiratory disease (*n* = 26, 4.7%) and diabetes mellitus (*n* = 22, 4%). A minority (*n* = 21, 3.8%) of the participants had a self-reported history of COVID-19 infection before their first vaccination dose. Most participants were patient-facing HCWs such as nurses (49.6%), doctors (14.3%), assistant medical officers (9.8%) and pharmacists (8.2%). There were 310 (56%) participants who reported at least one occupational exposure to SARS-CoV-2, with the most common exposures being face-to-face exposure (*n* = 192, 35%), providing care to COVID-19 patients (*n* = 162, 29%) and having direct contact with the COVID-19 environment (*n* = 162, 29%). Almost all (95–97%) complied with COVID-19 standard operating procedures at the workplace and social activities.

**Table 1.** Baseline demographic.

	Total (*n* = 551)	HKL (*n* = 300)	HQE (*n* = 130)	HQE2 (*n* = 121)
Age in years (mean (SD))	33.6 (7.2)	32.9 (6.5)	32.5 (6.0)	36.8 (8.9)
Gender (female)	417 (75.7%)	211 (70.3%)	108 (83.1%)	98 (81.0%)
BMI (mean (SD))^a^	27.1 (5.8)	27.4 (5.9)	26.8 (6.2)	26.6 (4.9)
*BMI category*^a^				
Underweight	14 (2.5%)	8 (2.7%)	4 (3.1%)	2 (1.7%)
Normal	213 (38.7%)	113 (37.7%)	51 (39.2%)	49 (40.5%)
Overweight	177 (32.1%)	89 (29.7%)	43 (33.1%)	45 (37.2%)
Obese	145 (26.3%)	89 (29.7%)	32 (24.6%)	24 (19.8%)
*Ethnicity*				
Malay	263 (47.7%)	232 (77.3%)	18 (13.8%)	13 (10.7%)
Chinese	58 (10.5%)	27 (9.0%)	14 (10.8%)	17 (14.1%)
Indian	33 (6.0%)	25 (8.3%)	4 (3.1%)	4 (3.3%)
Indigenous people of Sabah and Sarawak	195 (35.4%)	14 (4.7%)	94 (72.3%)	87 (71.9%)
Other ethnic groups^b^	2 (0.4%)	2 (0.7%)	0 (0.0%)	0 (0.0%)
Had COVID-19 infection prior to vaccination	21 (3.8%)	7 (2.3%)	10 (7.7%)	4 (3.3%)
*Comorbidity*				
None	452 (82.0%)	243 (81.0%)	113 (86.9%)	96 (79.3%)
1	76 (13.8%)	47 (15.7%)	12 (9.23%)	17 (14.0%)
≥2	23 (4.2%)	10 (3.33%)	5 (3.85%)	8 (6.6%)
*Comorbidities*				
Hypertension	34 (6.2%)	14 (4.7%)	6 (4.6%)	14 (11.6%)
Diabetes mellitus	22 (4%)	12 (4.0%)	3 (2.3%)	7 (5.8%)
Dyslipidaemia	11 (2.0%)	8 (2.7%)	1 (0.8%)	2 (1.7%)
Allergy	28 (5.1%)	17 (5.7%)	4 (3.1%)	7 (5.8%)
Respiratory disease	26 (4.7%)	15 (5.0%)	8 (6.2%)	3 (2.5%)
*Occupation*				
Nurse	273 (49.6%)	159 (53.0%)	63 (48.5%)	51 (42.1%)
Doctor	79 (14.3%)	42 (14.0%)	17 (13.1%)	20 (16.5%)
Assistant Medical Officer	54 (9.8%)	38 (12.7%)	5 (3.8%)	11 (9.1%)
Pharmacist	45 (8.2%)	27 (9.0%)	8 (6.1%)	10 (8.3%)
Laboratory personnel	26 (4.7%)	11 (3.7%)	13 (10%)	2 (1.7%)
Other occupations^c^	74 (13.4%)	23 (7.6%)	24 (18.5%)	27 (22.3%)
*Department*				
Internal medicine	162 (29.4%)	84 (28.0%)	37 (28.5%)	41 (33.9%)
Surgery	101 (18.3%)	70 (23.3%)	17 (13.1%)	14 (11.6%)
Pharmacy	49 (8.9%)	27 (9.0%)	9 (6.9%)	13 (10.7%)
Anaesthesiology & Intensive Care	38 (6.9%)	16 (5.3%)	11 (8.5%)	11 (9.1%)
Pathology	34 (6.2%)	14 (4.7%)	15 (11.5%)	5 (4.1%)
Supporting Services	27 (4.9%)	10 (3.3%)	7 (5.4%)	10 (8.3%)
Accident & Emergency	24 (4.4%)	19 (6.3%)	0 (0.0%)	5 (4.1%)
Other departments^d^	116 (21.1%)	60 (20.0%)	34 (26.1%)	22 (18.2%)
*Occupational exposure to SARS-CoV-2*				
Perform nasopharyngeal swabbing	101 (18%)	73 (24%)	12 (9%)	16 (13%)
Face-to-face exposure (with or without PPE)	192 (35%)	122 (41%)	50 (39%)	20 (17%)
Provide care to COVID-19 patients	162 (29%)	100 (33%)	50 (39%)	12 (10%)
Perform aerosol-generating procedure	84 (15%)	52 (17%)	26 (20%)	6 (5%)
Handling biospecimen of COVID-19 patients	139 (25%)	83 (28%)	45 (35%)	11 (9%)
Contact with COVID-19 environment	162 (29%)	109 (36%)	39 (30%)	14 (12%)

HKL, Hospital Kuala Lumpur; HQE, Hospital Queen Elizabeth; HQE2, Hospital Queen Elizabeth 2; SD, standard deviation, BMI, body mass index.

^a^ BMI data were summarized from 549 participants. Two participants did not provide height and weight for BMI calculation.

^b^ Other ethnic groups include Semai and Siamese.

^c^ Other occupations include health attendant, radiographer/X-ray technician, physiotherapist/occupational therapist, dietitian/nutritionist, administrative staff, dentist, health inspector, disinfection team, driver.

^d^ Other departments include orthopaedics, radiology, paediatric, ophthalmology, obstetrics and gynaecology, state health department, psychiatry, otolaryngology, administrative office, dental, cleaning services, and primary care.

### Anti-S1-RBD antibody titre over 24 weeks

**Figure 1.**
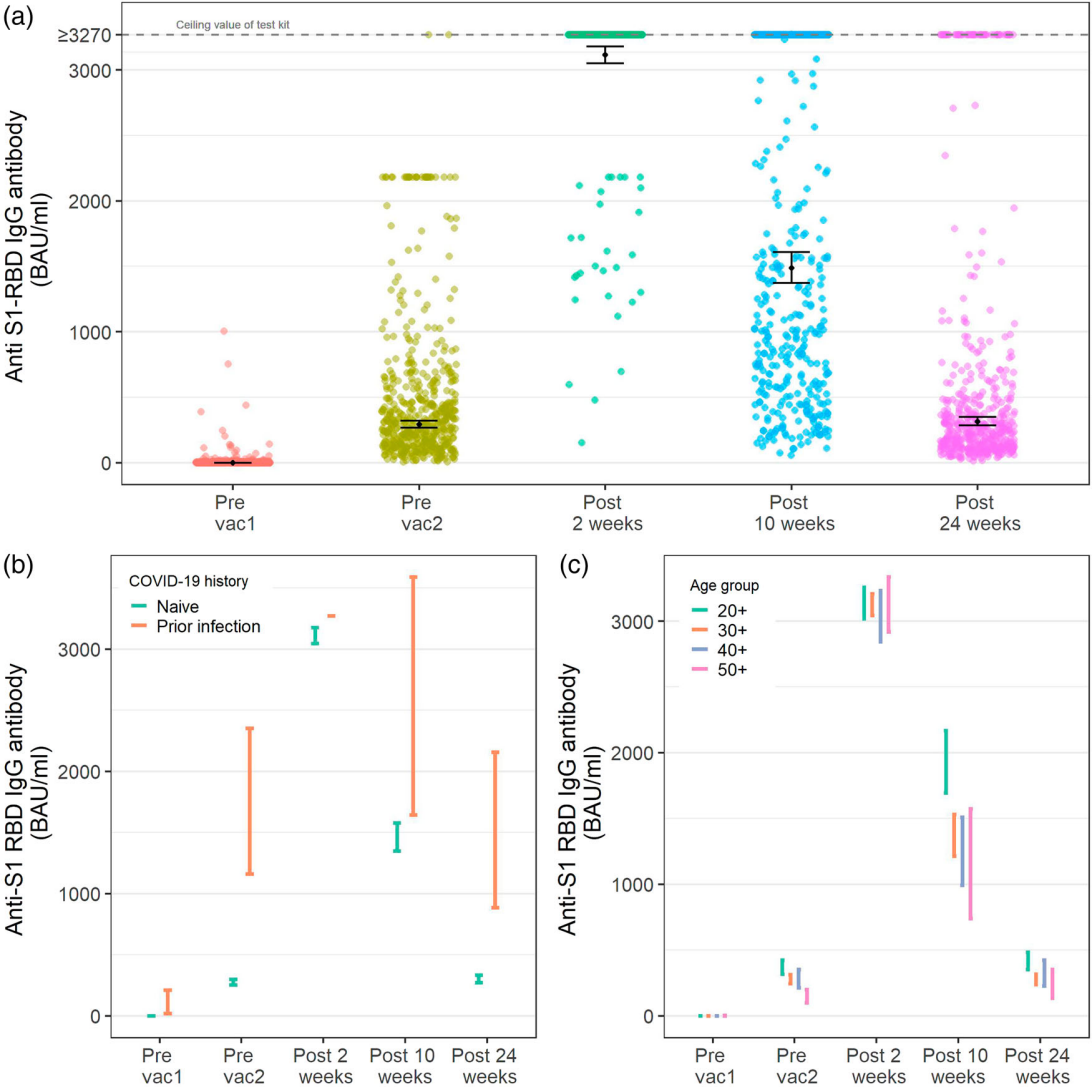
Longitudinal anti-S1-RBD IgG antibody response and its associated factors. (a) The anti-S1-RBD IgG antibody response over 6 months. (b) The anti-S1-RBD IgG antibody response was stratified by COVID-19 infection status prior to vaccination. (c) The anti-S1-RBD IgG antibody response was stratified by age groups. Pre-vac1, up to 3 days before the first dose of vaccine (*n* = 551); pre-vac2, up to 3 days before the second dose of vaccine (*n* = 527); post-2, 2 weeks after the second vaccine (*n* = 515); post-10 weeks, 10 weeks after the second vaccine (*n* = 519); post-24 weeks, 24 weeks after the second vaccine (*n* = 499). The dots and error bars in the figures indicate the geometric mean titre and its 95% confidence interval.

At baseline, 26 (4.7%) participants were seropositive with detectable reactive anti-S1-RBD antibodies. Subsequently, the seropositivity rates remained high at 98.5%, 100%, 100% and 99.6% at before second vaccine, 2, 10 and 24 weeks, respectively. At 2 weeks after the second dose, all participants had antibody titre levels consistent with recent vaccination, with the GMT close to the maximum detectable value at 3115 BAU/ml (95%CI, 3051–3180). In 10 weeks, the GMT reduced by 54.6% to 1486 BAU/ml (95% CI, 1371–1610), and by 24 weeks, the GMT declined by 90.3% to 315 BAU/ml (95% CI, 283–349). (Figure 1(a) and Table 2)

**Table 2.** The geometric mean of anti-S1-RBD IgG titre (GMT) at each visit, stratified according to the covariates found significant in the linear mixed model.

Variables	Pre-vaccine 1 (*n *= 551)		Pre-vaccine 2 (*n *= 527)		Post-2 weeks (*n *= 515)		Post-10 weeks (*n *= 519)		Post-24 weeks (*n *= 499)	
	GMT(95%CI)	*P*	GMT (95%CI)	*P*	GMT (95%CI)	*P*	GMT (95%CI)	*P*	GMT (95%CI)	*P*
Seropositive (*n*, %)	26 (4.7%)	–	519 (98.5%)	–	515 (100%)	–	519 (100%)	–	497 (99.6%)	–
Overall GMT	0 (0–0)	–	293 (268–321)	**<0**.**001**	3115 (3051–3180)	**<0**.**001**	1486 (1371–1610)	**<0**.**001**	315 (283–349)	**<0**.**001**
*COVID-19 infection before vaccination*										
No	0 (0–0)	**<0**.**001**	275 (252–300)	**<0**.**001**	3109 (3045–3174)	0.303	1458 (1347–1579)	**0**.**001**	300 (271–331)	**<0**.**001**
Yes	60 (17–209)		1652 (1161–2350)		3270 (3270–3270)		2429 (1644–3589)		1380 (883–2156)	
*Age group*										
20+	0 (0–0)	0.191	365 (315–424)	**<0**.**001**	3135 (3017–3257)	0.549	1916 (1694–2168)	**<0**.**001**	412 (351–483)	**<0**.**001**
30+	0 (0–0)		276 (244–313)		3124 (3044–3206)		1363 (1214–1529)		275 (239–316)	
40+	0 (0–0)		274 (214–352)		3031 (2845–3230)		1223 (993–1507)		310 (228–423)	
50+	0 (0–2)		140 (98–200)		3120 (2919–3334)		1078 (739–1571)		218 (136–350)	
*BMI category*										
Underweight	0 (0–0)	0.673	504 (350–725)	0.103	3270 (3270–3270)	0.577	2367 (1723–3251)	**0**.**040**	754 (405–1403)	0.083
Normal	0 (0–0)		283 (246–325)		3143 (3039–3251)		1614 (1426–1826)		318 (273–372)	
Overweight	0 (0–0)		279 (241–324)		3060 (2946–3178)		1448 (1266–1655)		307 (259–365)	
Obese	0 (0–0)		313 (259–377)		3128 (3022–3237)		1304 (1108–1535)		294 (240–361)	
*Comorbidity*										
0	0 (0–0)	0.281	295 (268–325)	0.167	3131 (3066–3197)	0.670	1527 (1402–1663)	**0**.**049**	320 (287–357)	0.401
1	0 (0–0)		316 (250–401)		3020 (2809–3247)		1449 (1167–1798)		312 (239–407)	
≥2	0 (0–1)		208 (137–316)		3118 (2923–3326)		953 (630–1442)		235 (136–406)	
*Breakthrough infection*										
No	0 (0–0)	0.477	301 (274–330)	0.259	3124 (3058–3191)	0.711	1504 (1386–1633)	0.485	254 (233–278)	**<0**.**001**
Yes	0 (0–0)		233 (177–307)		3039 (2863–3226)		1340 (1033–1738)		2038 (1547–2685)	** **

GMT, geometric mean titre; BMI, body mass index; pre-vaccine 1, up to 3 days before receiving the first dose of vaccine; pre-vaccine 2, up to 3 days before receiving the second dose of vaccine; post-2, 2 weeks after the second vaccine; post-10, 10 weeks after the second vaccine; post-24, 24 weeks after the second vaccine.

Significant value for overall GMT at each visit is obtained from the linear mixed model using “visit” as the only covariate and baseline visit as the reference group. The first row of each categorical covariate is the reference group; except for the BMI category, the reference is normal BMI. Mann-Whitney U test was used to test covariates with two groups; Kruskal-Wallis nonparametric test was used to test covariates with more than two groups. The bold font indicates significant difference (*p* < 0.05) compared to the reference group.

### Anti-S1-RBD antibody titre stratified by covariates

An analysis from the linear mixed model identified COVID-19 infection history, age groups and breakthrough infection as having significant effects (*p *< 0.001) on the kinetics of anti-S1-RBD antibody titre over time, BMI category and comorbidities only showed a marginal effect on antibody titre (Table 2 and Supplementary Table 1). Participants who had prior COVID-19 infection had GMT levels 1.6 to 6 times higher than those who were infection naïve. (Figure 1(b) and Table 2) HCWs in the 20–29 years age group constantly had higher GMT than older age groups; this was especially prominent at 10 weeks after vaccination (1916 BAU/ml vs. 1078 BAU/ml, *p* < 0.001). (Figure 1(c) and Table 2) This age effect, however, diminished at 24 weeks post-vaccination.

### Breakthrough infections and anti-S1-RBD antibody

Post-vaccination, one case of breakthrough infection occurred before 10 weeks, while 56 breakthrough cases (10%) occurred between 10 and 24 weeks. Of the 56 cases, 3, 20, 27 and 6 cases occurred at 3-, 4-, 5- and 6-month post-vaccination, respectively. For these cases, 45 (80%) presented mild symptoms without pneumonia, and 11 (20%) were asymptomatic cases detected during contact tracing. Of the 45 mildly symptomatic participants, the six most common symptoms were fever (*n* = 34, 75.5%), runny nose (*n* = 31, 68.9%), cough (*n* = 29, 64.4%), loss of smell (*n* = 28, 62.2%), headache (*n* = 24, 53.3%) and sore throat (*n* = 23, 51.1%). All breakthrough cases did not require hospitalization. Interestingly, none of the convalescents vaccinated participants had a breakthrough infection. More breakthrough cases occurred in the Malay ethnic group, among nurses and those working in the surgery and internal medicine departments. (Supplement Table 2) We found no significant difference when comparing the demographic and occupational exposure variables of breakthrough and non-breakthrough individuals (Supplement Table 2).

**Figure 2.**
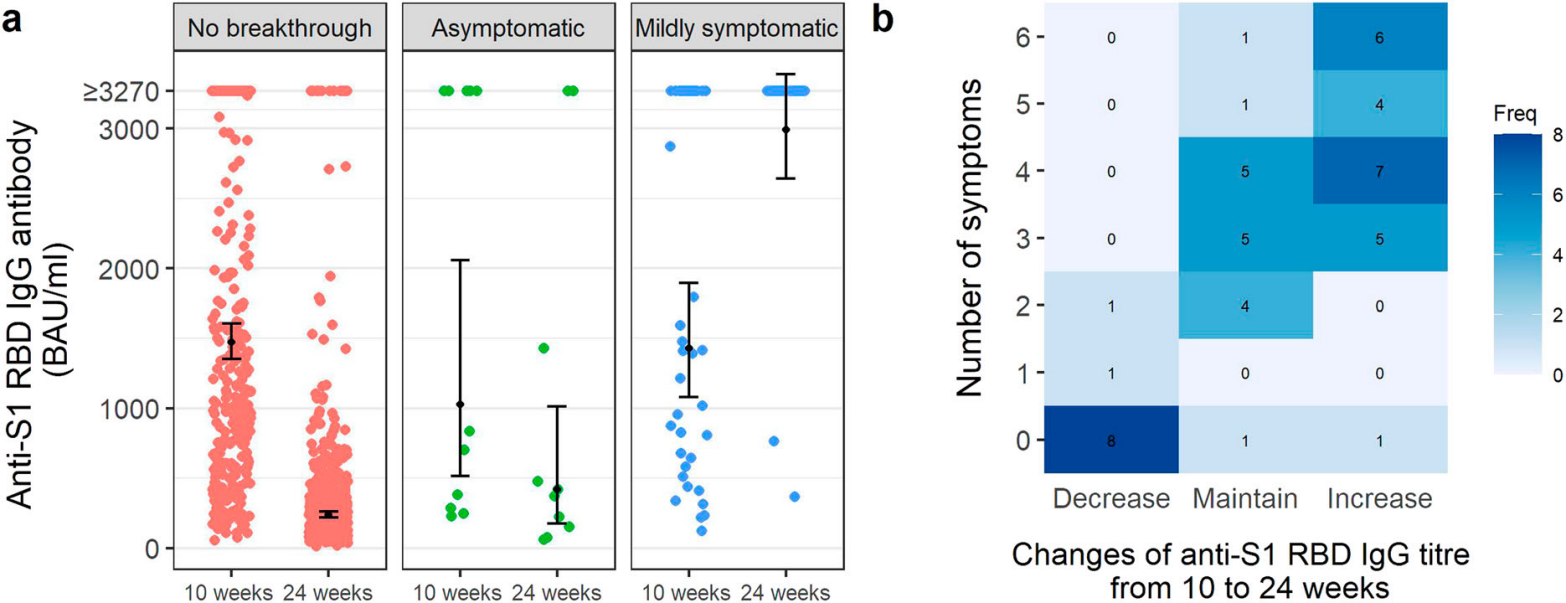
GMT stratified by breakthrough infection and clinical category. (a) GMT of anti-S1-RBD antibody stratified by breakthrough infection and clinical category. At peri-infection (10 weeks), the GMT of anti-S1-RBD IgG antibody is not significantly different across the three groups. (Kruskal-wallis test, *p* = 0.597) At post-infection, the GMT of anti-S1-RBD IgG antibody is significantly different across the group. (Kruskal-wallis test, *p *< 0.001). Post-hoc Dunn’s test showed a significant difference between the no breakthrough group and the mildly symptomatic group (*p *< 0.001) and between the asymptomatic group and the mildly symptomatic group (*p *< 0.001). (b) The relationship between the number of symptoms experienced by breakthrough individuals and the kinetics of anti-S1-RBD antibody. The symptoms include fever, headache, cough, runny nose, sore throat, and loss of smell.

At 10 weeks (before the surge of breakthrough infections), the GMT was not different between the breakthrough and non-breakthrough individuals; at 24 weeks (after the surge of infections), the GMT was significantly higher in individuals with a record of breakthrough infection (2038 BAU/ml [95%CI, 1547–2685]) compared to those without (254 BAU/ml [95%CI, 233–278]). (Table 2). By stratifying the infections and clinical category, at 24 weeks, the post-breakthrough GMT among symptomatic individuals was significantly higher than those who were asymptomatic and uninfected (2991 BAU/ml vs. 422 BAU/ml vs. 236 BAU/ml, *p *< 0.001). (Figure 2(a)) Anti-S1-RBD antibodies rose sharply or maintained above ceiling value in 95% of the mildly symptomatic individuals. In contrast, antibody levels waned in 80% of the asymptomatic individuals, an observation similar to the uninfected individuals. (Figure 2(a,b)) The number of symptoms correlated with the likelihood of increased anti-S1-RBD antibody titre post-infection (odds ratio = 5.78 (95%CI 2.55, 22.3), *p* < 0.001).

## Discussions

This study provides an insight into the immunogenicity of BNT162b2 vaccination among ethnically-diverse HCWs in Malaysia. The GMT of anti-S1-RBD antibodies declined significantly after 10–24 weeks (i.e. approximately 3–6 months) post-vaccination. Besides time since vaccination, individual characteristics, such as past infection and age, significantly influenced the antibody titre levels. The clinical data concurred with immunological data, where a spike in breakthrough infections at 10–24 weeks post-vaccination is noticed alongside waning antibody titre levels. The GMT of the breakthrough and non-breakthrough participants did not differ significantly before breakthrough infection. Participants with asymptomatic breakthrough infection did not develop robust antibody response after that, but those with at least mild symptoms had their antibody titre levels boosted.

### Breakthrough infection

About 10% of our participants developed breakthrough infection between 10 and 24 weeks after BNT162b2 vaccination, a finding similar to that reported by Malaysia National Occupational Health and Safety Surveillance, where 8% of the 280,000 vaccinated HCWs contracted breakthrough infection up to 7 months after the launch of the national vaccination programme. However, the breakthrough rate was substantially higher for BNT162b2 or mRNA vaccine recipients in other countries [15]. One reason could be the waning of vaccine effectiveness over time [16]. Studies with limited observation periods of 2 weeks to 3 months post-vaccination, captured fewer breakthrough cases as immunity markers were still heightened. In contrast, the spike of breakthrough infections in our study tallied with the substantial reduction in antibody responses from 10 weeks onwards. Another plausible explanation was that our observation period coincided with the emergence and dominance of the Delta variant in Malaysia from July 2021 [4,17]. Our study findings were consistent with the reported reduced neutralization capacity of vaccine-induced antibody towards Delta strain spike protein 3 months after vaccination [18]. During this period, despite strict compliance to protocols of personal protective equipment, HCW participants were more exposed to the SARS-CoV-2 virus via healthcare procedures and close contact from and outside work. (Table 3) Taken together, the high breakthrough rate among HCWs is explained by the waning of antibody response coupled with the emergence of the more infectious Delta variant that led to higher COVID-19 incidence in the general population. Nevertheless, all breakthrough infections were mild or asymptomatic; the primary vaccination with BNT162b2 is still highly protective against severe infection for up to 6 months.

**Table 3.** Descriptive statistics of variables and clinical outcomes collected at each visit.

	Pre-vaccine 1 (*n* = 551)	Post-2 weeks (*n* = 515)	Post-10 weeks (*n* = 519)	Post-24 weeks (*n* = 499)
*Potential exposure to SARS-CoV-2*				
At least 1 occupational-related exposure	310 (56%)	138 (27%)	196 (38%)	285 (57%)
*List of occupational-related exposure*				
Perform nasopharyngeal swabbing	101 (18%)	37 (7%)	49 (9%)	94 (19%)
Face-to-face exposure (with or without PPE)	192 (35%)	67 (13%)	130 (25%)	178 (36%)
Provide care to COVID-19 patients	162 (29%)	67 (13%)	123 (24%)	177 (35%)
Perform aerosol-generating procedure	84 (15%)	35 (7%)	62 (12%)	88 (18%)
Handling biospecimen of COVID-19 patients	139 (25%)	69 (13%)	94 (18%)	138 (28%)
Contact with COVID-19 environment	162 (29%)	66 (13%)	105 (20%)	157 (31%)
*Close contact to COVID-19 case*^a^				
At work	–	18 (3%)	69 (13%)	179 (36%)
Outside work	–	0 (0%)	14 (3%)	49 (10%)
*Compliance to SOP*^b^				
At workplace	520 (95%)	481 (93%)	496 (96%)	474 (95%)
At public places^b^	530/546 (97%)	487/513 (95%)	498/511 (97%)	480/492 (98%)
During social events^b^	530/546 (97%)	488/515 (95%)	500/513 (97%)	484/495 (98%)
*Clinical outcomes*				
Had symptoms in the past 2 weeks	–	195 (38%)	37 (7%)	70 (14%)
Had breakthrough infection in-between visits	–	–	1 (0.2%)	56 (10%)^d^
Asymptomatic	–	–	1/1 (100%)	11/56 (20%)
Mild symptoms	–	–	0/1 (0%)	45/56 (80%)
*Presumed source of infection*^c^				
Handling COVID-19 patients at work	–	–	–	16/56 (28%)
From healthcare colleagues	–	–	–	11/56 (20%)
From household members	–	–	–	15/56(27%)
From community or friends (not from the same household)	–	–	–	5/56 (9%)
Unknown	–	–	–	9/56 (16%)

PPE, personal protection equipment, SOP, standard operating procedures.

^a^ Close contact and symptoms data were collected only after complete vaccination.

^b^ Some participants did not visit public places or attend social events within 1 month when prompted to answer compliance questions. Hence, the compliance to SOP questions did not apply to these participants. The number of participants, who answered compliance, is noted as the denominator.

^c^ Self-reported presumed source of infection for the 56 breakthrough infections that occurred between 10 and 24 months post-vaccination.

^d^ There were 3, 20, 27 and 6 breakthrough infections that occurred at 3-, 4-, 5-, and 6-month post-vaccination, respectively.

### Correlation of protection

We did not observe a cut-off from pre-breakthrough infection antibody titre that discriminates the risk of infection at an individual level. This study found no difference in the pre-infection anti-S1 RBD antibody titres of breakthrough and non-breakthrough individuals. Moreover, 45% of our breakthrough cases happened when their 10-week antibody titres were still beyond the upper threshold of the assay kit (>3270 BAU/ml). A high antibody titre does not guarantee absolute protection towards mild and asymptomatic breakthroughs. This finding is similar to Ferrari et al. and Eyre et al., albeit with more minor breakthrough incidences [19,20]. Inactivated SARS-CoV-2 vaccine study found that individuals with the evidence of neutralizing capability were still infected with mild disease [21]. A case–control study on BNT162b2 HCW vaccinees found breakthrough infections were more correlated with neutralizing antibody titres than binding IgG titres [22]. New evidence from the mRNA-1273 COVID-19 vaccine trial suggests incremental risk when antibody titre falls but could not provide a binary cut-off [23]. The sudden increase in mild breakthrough cases observed in our study might be due to the highly transmissible variant circulating in the community [24]. Even though the utility of individual IgG antibody titre in determining the correlation of protection against infection remains unclear, the antibody titre at such a level seems to ward off moderate and severe infection.

### Hybrid immunity of convalescents

Convalescent vaccinated individuals consistently have higher and more durable IgG titres than uninfected vaccinated individuals up to 6 months post--vaccination. This confirms that the immune responses of convalescent individuals triumph over that of naïve individuals with more robust antibody and memory B cells’ response and better neutralization potency after vaccination [25,26]. Also, no convalescent vaccinated individuals developed a breakthrough infection. We speculate that hybrid immunity from natural infection and vaccination offers longer and better protection against the Delta variant. As the pandemic and vaccination programmes evolve, the discussion on hybrid immunity against Omicron is of growing importance.

### Symptoms of breakthrough infections and booster vaccination

Most intriguing, our study found that post-breakthrough anti-S1-RBD antibodies only increased among the symptomatic but not the asymptomatic individuals. The likelihood of heightened antibody levels also increased with the number of symptoms experienced. Before COVID-19 vaccines became widely available, evidence showed that the magnitude of immunity response of the naturally infected people was associated with disease severity and symptoms and that asymptomatic individuals elicited weaker immune response and were more likely to revert seronegative [27–29]. We speculate primary vaccination mitigated the severity of the asymptomatic breakthrough to only localized infections that were cleared off by innate immunity at mucosal surfaces without triggering an adaptive immune response. By contrast, symptomatic breakthroughs resulted in heightened humoral responses that may act as natural boosters. A more extended observation period on symptomatic breakthrough individuals is needed to understand the kinetics and protection from such “hybrid immunity.” A recent laboratory study showed that Omicron was susceptible to robust neutralization from serum of breakthrough individuals but evaded neutralization by vaccinated non-breakthrough individuals [30]. An epidemiology report on Omicron from the UK demonstrated low protection by primary vaccination but moderate-to-high protection by booster vaccination [10]. Taken together, our finding supports administering booster vaccine to the vaccinated individuals without a breakthrough and vaccinated individuals with asymptomatic breakthrough to improve immunity against Omicron. However, the timing of boosters for symptomatic breakthrough individuals may warrant separate consideration.

### Limitations and strengths

There are some limitations to our study. We did not assess neutralizing activity against different VOCs due to resource restrictions. Nevertheless, the immunoassay manufacturer’s report claimed that the measured anti-S1-RBD titre is highly correlated with the PRNT_50_ virus neutralization titre. Second, our cohort consists of younger HCWs with few health comorbidities but higher exposure risk; readers are cautioned not to generalize the results to the older population and immunocompromised groups. Lastly, this study only sampled HCWs who received the BNT162b2 vaccine; as Malaysia has since included more vaccine brands into the vaccination programme, there is a need to expand this surveillance to include other COVID-19 vaccines and samples from the wider population.

The strength of this study is the inclusion of ethnically-diverse HCWs in Malaysia and be representative of other Asian countries composed of similar ethnic groups. We addressed the issue of Asian populations being under-represented in BNT162b2 clinical trials and longitudinal research, confirmed the high immunogenicity and adequate durability of BNT162b2-induced immunity, and supported the widespread use of BNT162b2 among the Asian population. Second, collecting serial blood samples and clinical surveillance data with low drop-out rates enabled us to supplement and interpret epidemiology data with laboratory findings. Third, while sampling from HCWs, more than one-third contracted the infection from household members and communities. (Table 3). Therefore, the study results can be carefully postulated to the general public vaccinated for BNT162b2.

## Conclusion

BNT162b2 produced a high immunogenic response in Asian ethnics. Although protection against mild breakthrough infection declined over time with waning antibodies and emerging variants, the benefits against severe disease are preserved. After breakthrough infection, individuals with symptomatic presentation elicited strong immune responses. The study findings support offering booster vaccination for BNT162b2 recipients, including those who recovered from asymptomatic breakthrough infection.

## Supplementary Material

Supplemental Material
