# Supplementary material for: COVID-19 breakthrough infections and humoral immune response among BNT162b2 vaccinated healthcare workers in Malaysia
Source: Emerg Microbes Infect. 2022 May 3;11(1):1262–71. doi: 10.1080/22221751.2022.2065936 (PMC9067955; doi:10.1080/22221751.2022.2065936)
Supplement: Supplemental Material [file TEMI_A_2065936_SM3083.docx]

**Supplementary material**

**Supplementary methods and material.** Supplemental information on self-administered questionnaires.

**Supplementary Table 1.** Linear mixed model of anti-S1-RBD IgG antibody titre

**Supplementary Table 2.** Comparison of demographics data and occupational exposure of breakthrough and non-breakthrough individuals

**Supplementary methods and material.** Supplemental information on self-administered questionnaires.

At baseline visit, consented participants answered a self-administered questionnaire which comprised of four sections: (A) sociodemographic data, (B) exposure to SARS-CoV-2 at workplace, (C) adherence to infection prevention and control, and (D) behavioural risk assessment.

In the sociodemographic sections, participants were asked to fill up date of birth (to calculate age), sex, self-identified ethnicity, comorbidities, height, weight, profession, workplace and department and previous infection with COVID-19.

To assess exposure risk, we asked if participants were exposured to SARS-CoV-2 via these six types of occupational procedures in the past month. The procedures include, (i) performing oropharyngeal/nasopharyngeal swabbbing, (ii) face-to-face exposure within 1 meter with a confimed COVID-19 cases (with or without PPE), (iii) provide care to a COVID-19 patient, (iv) performing aerosolizing procedures, (v) handling body fluids or biospecimens of a confirmed COVID-19 patient, and (vi) direct contact with the environments around confirmed COVID-19 case. These questions were adapted from a risk assessment tool developed by the WHO.^1^

To assess adherence to infection prevention and control, we asked participants to quantify the frequency they comply with the personal protective equipment (PPE) usage guidelines and hand hygiene when performing procedures (i), (iii), (iv) and (v). Participants were asked to self-rate their adherence with four choices: ‘Always, as recommended’ – defined as more than 95% of the time; ‘most of the time’ – defined as 50% or more, but not 100% of the time; ‘occasionally’ – defined as 20% to 50% of the time; and ‘rarely’ – defined as less than 20% of the time. These items were adapted from a risk assessment tool developed by the WHO.^1^

To assess behavioral risk, participants were asked to indicate if they adhere to the new normal when they are at workplace, public places and while attending social gathering. ‘Always, as recommended’ – defined as more than 95% of the time; ‘most of the time’ – defined as 50% or more, but not 100% of the time; ‘occasionally’ – defined as 20% to 50% of the time; and ‘rarely’ – defined as less than 20% of the time. These questions were adapted from MyRisk tool developed by the Institute for Behavioural Research and risk assessment tool by the WHO.^1,2^

Reference

1. World Health Organization. *Health Workers Exposure Risk Assessment and Management in the Context of COVID-19 Virus*.; 2020. https://apps.who.int/iris/handle/10665/331340.

2. myRisk. http://myrisk.nih.gov.my/. Published 2020.

**Supplementary Table 1.** Linear mixed model of anti-S1-RBD IgG antibody titre

| (1) Examine the association of covariates with kinetics of anti S1-RBD IgG antibody titre.  - Testing 1 covariate at one time.  - For example: **antibody titre ~ visit + 1 covariate + (1\| subject_ID)** | | | | | |
| --- | --- | --- | --- | --- | --- |
|  | ꞵ | Std. Error | *t* statistics | df | *P* value |
| Visit 2 | 480.79 | 44 | 10.928 | 2097 | **<0.001** |
| Visit 3 | 3162.14 | 44.29 | 71.404 | 2105 | **<0.001** |
| Visit 4 | 2035 | 44.19 | 46.051 | 2103 | **<0.001** |
| Visit 5 | 682.56 | 44.69 | 15.275 | 2113 | **<0.001** |
| Prior infection | 786.64 | 96.13 | 8.183 | 555 | **<0.001** |
| Age.cat30+ | -173.19 | 41.63 | -4.161 | 556 | **<0.001** |
| Age.cat40+ | -187.22 | 60.07 | -3.116 | 542 | **0.002** |
| Age.cat50+ | -305.7 | 92.65 | -3.3 | 530 | **0.001** |
| BMI underweight | 271.73 | 123.35 | 2.203 | 561 | **0.028** |
| BMI overweight | -53.07 | 44.78 | -1.185 | 547 | 0.24 |
| BMI obese | -42.11 | 47.44 | -0.888 | 548 | 0.38 |
| Comorbidity - 1 | -23.76 | 54.7 | -0.434 | 548 | 0.66 |
| Comorbidity - ≥ 2 | -178.6 | 93.73 | -1.906 | 538 | 0.057 |
| Sex | 39.33 | 44.05 | 0.893 | 552 | 0.37 |
| Breakthrough infection | 355.95 | 60.21 | 5.912 | 545 | **<0.001** |
|  | | | | | |
| (2) Examine the best model by fitting with only the significant covariates in it and finalized model with the smallest AIC value.  **Best model: antibody titre ~ visit * breakthrough + Prior infection +age category+(1\| subject_ID)** | | | | | |
|  | ꞵ | Std. Error | *t* value | df | *P* value |
| (Intercept) | 96.66 | 39.04 | 2.476 | 1522 | **0.013** |
| Visit 2 | 496.03 | 41.83 | 11.859 | 2099 | **<0.001** |
| Visit 3 | 3172.74 | 42.16 | 75.249 | 2109 | **<0.001** |
| Visit 4 | 2049.44 | 42.06 | 48.726 | 2107 | **<0.001** |
| Visit 5 | 457.51 | 42.48 | 10.769 | 2116 | **<0.001** |
| Breakthrough | 22.51 | 98.12 | 0.229 | 2452 | 0.82 |
| Prior infection | 834.06 | 89.85 | 9.283 | 558 | **<0.001** |
| Age.cat30+ | -165.96 | 37.39 | -4.439 | 557 | **<0.001** |
| Age.cat40+ | -196.88 | 53.95 | -3.649 | 543 | **<0.001** |
| Age.cat50+ | -318.65 | 83.36 | -3.822 | 531 | **<0.001** |
| Visit 2: Breakthrough | -128.5 | 130.77 | -0.983 | 2091 | 0.33 |
| Visit 3: Breakthrough | -69.08 | 130.28 | -0.53 | 2087 | 0.60 |
| Visit 4: Breakthrough | -111.14 | 130.25 | -0.853 | 2088 | 0.39 |
| Visit 5: Breakthrough | 2264.01 | 132.88 | 17.038 | 2107 | **<0.001** |
| Random effect: ~ (1\| Subject_ID)  Variance (sd) of random effect: 84433 (249)  Variance (sd) of residual: 519096 (649)  Intraclass correlation (ICC): 0.14  Model Fit: AIC = 41514 | | | | | |

The references of categorical variables are as follow:

The reference group for visit variable is visit 1 (baseline visit before receive first dose vaccine).

The reference group for age category is 20+ (20-29) years old.

The reference group for BMI is normal BMI.

The reference group for comorbidity is no comorbidity.

The reference group for sex is male.

**Supplementary table 2.** Comparison of demographics data and occupational exposure of breakthrough and non-breakthrough individuals

|  | **Breakthrough cases**  **(n=56)** | **Non-breakthrough**  **(n=493)** | ***p* value** |
| --- | --- | --- | --- |
| Age in years (mean (SD)) | 33.02 (6.28) | 33.73 (7.27) | 0.483 |
| Gender female | 40 (71.4) | 375 (76.1) | 0.548 |
| Ethnicity |  |  | 0.087 |
| Malay | 35 (62.5) | 227 (46.0) |  |
| Chinese | 3 ( 5.4) | 54 (11.0) |  |
| Indian | 5 ( 8.9) | 28 ( 5.7) |  |
| Indigenous people of Sabah and Sarawak | 13 (23.2) | 182 (36.9) |  |
| Other ethnic groups^a^ | 0 ( 0.0) | 2 ( 0.4) |  |
| BMI (mean (SD)) | 26.20 (6.08) | 27.18 (5.74) | 0.233 |
| COVID-19 infection before vaccination | 0 ( 0.0) | 20 ( 4.1) | 0.246 |
| Occupation |  |  | 0.317 |
| Nurse | 31 (55.4) | 242 (49.1) |  |
| Assistant medical officer | 8 (14.3) | 46 ( 9.3) |  |
| Doctor | 5 ( 8.9) | 72 (14.6) |  |
| Pharmacist | 2 ( 3.6) | 43 ( 8.7) |  |
| Other occupations^b^ | 10 (17.9) | 90 (18.3) |  |
| Department |  |  | 0.011 |
| Surgery | 18 (32.1) | 83 (16.8) |  |
| Internal medicine | 16 (28.6) | 146 (29.6) |  |
| Anaesthesiology & Intensive Care | 6 (10.7) | 31 ( 6.3) |  |
| Accident & Emergency | 5 ( 8.9) | 19 ( 3.9) |  |
| Pharmacy | 2 ( 3.6) | 47 ( 9.5) |  |
| Supporting services | 2 ( 3.6) | 25 ( 5.1) |  |
| Pathology | 1 ( 1.8) | 33 ( 6.7) |  |
| Other departments^c^ | 6 (10.7) | 109 (22.1) |  |
| Comorbidities |  |  | 0.867 |
| None | 46 (82.1) | 404 (81.9) |  |
| 1 | 7 (12.5) | 69 (14.0) |  |
| ≥2 | 3 ( 5.4) | 20 ( 4.1) |  |
| Had at least 1 occupational exposure^d^ | 32 (62.7) | 253 (56.5) | 0.479 |
| Perform nasopharyngeal swabbing | 13 (25.5) | 81 (18.1) | 0.419 |
| Face-to-face exposure (with or without PPE) | 17 (33.3) | 161 (35.9) | 0.727 |
| Provide care to COVID-19 patients | 20 (39.2) | 157 (35.0) | 0.473 |
| Perform aerosol-generating procedure | 12 (23.5) | 76 (17.0) | 0.299 |
| Handling biospecimen of COVID-19 patients | 16 (31.4) | 122 (27.2) | 0.570 |
| Contact with COVID-19 environment | 21 (41.2) | 136 (30.4) | 0.184 |

^a^Other ethnic groups include Semai and Siamese.

^b^Other occupations include lab personnel, health attendant, radiographer/ X-ray technician, physiotherapist/ occupational therapist, dietitian/ nutritionist, administrative staff, dentist, health inspector, disinfection team, driver.

^c^Other departments include orthopaedics, radiology, paediatric, ophthalmology, obstetrics & gynaecology, state health department, psychiatry, otolaryngology, administrative office, dental, cleaning services, primary care.

^d^Self reported occupational expsoure to SARS-CoV-2 between 10 to 24 weeks post vaccination.
